# Serological Markers Associated With Response to Immune Checkpoint Blockade in Metastatic Gastrointestinal Tract Cancer

**DOI:** 10.1001/jamanetworkopen.2019.7621

**Published:** 2019-07-24

**Authors:** Zhihao Lu, Jianling Zou, Ying Hu, Shuang Li, Tao Zhou, Jifang Gong, Jian Li, Xiaotian Zhang, Jun Zhou, Ming Lu, Xicheng Wang, Zhi Peng, Changsong Qi, Yanyan Li, Jie Li, Yan Li, Jianyin Zou, Xiao Du, Henghui Zhang, Lin Shen

**Affiliations:** 1 Department of Gastrointestinal Oncology, Key Laboratory of Carcinogenesis and Translational Research (Ministry of Education), Peking University Cancer Hospital & Institute, Beijing, China; 2 Genecast Precision Medicine Technology Institute, Beijing, China; 3 Institute of Infectious Diseases, Beijing Ditan Hospital, Capital Medical University, Beijing, China; 4 Department of Otolaryngology, Otolaryngology Institute of Shanghai Jiao Tong University, Shanghai Jiao Tong University Affiliated Sixth People’s Hospital, Shanghai, China

## Abstract

**Importance:**

There are a limited number of predictive biomarkers for hyperprogressive disease (HPD), which is induced by immune checkpoint blockade (ICB) therapy.

**Objective:**

To evaluate the association of biomarkers in serum with the response to ICB therapy in patients with metastatic gastrointestinal tract cancer.

**Design, Setting, and Participants:**

Cohort study in which patients with metastatic gastrointestinal tract cancer treated with ICB were enrolled at the Department of Gastrointestinal Oncology, Peking University Cancer Hospital and Institute, Beijing, China, from August 1, 2015, to July 31, 2017, with the last follow-up date on January 1, 2018. Serum samples were collected at baseline and during the first visit to the clinic after starting treatment. Data analysis was conducted from January 16, 2018, to September 1, 2018.

**Exposures:**

A total of 59 factors, including cytokines/chemokines, growth factors, and soluble checkpoint-related proteins in serum, were examined by multiplexed bead immunoassays.

**Main Outcomes and Measures:**

Tree-based estimators were used to evaluate the importance of serum protein levels to ICB treatment response. Progression-free survival and overall survival analyses were conducted with the Kaplan-Meier method and log-rank test.

**Results:**

In total, 56 patients were examined. All patients with HPD (5 [8.9%]) had significantly lower mean (SD) levels of serum monocyte chemoattractant protein 1 than patients without HPD at baseline (53.4 [17.3] pg/mL vs 106.4 [48.4] pg/mL; *P* = .02). All patients with HPD were also identified by lower leukemia inhibitory factor levels (<13.28 pg/mL) and higher cluster of differentiation 152 levels (≥31.81 pg/mL). Among the remaining 51 patients, responders with esophageal squamous cell carcinoma (ESCC) or colorectal cancer (CRC) showed larger decreases in interleukin 1 receptor antagonist levels than nonresponders (ESCC: −55.02% [95% CI, −86.52% to −23.51%] vs 43.44% [95% CI, 11.93% to 74.96%]; *P* < .001; CRC: −35.82% [95% CI, −67.38% to −4.26%] vs 59.14% [95% CI, −72.34% to 190.6%]; *P* = .04). Responders with gastric cancer (GC) had larger increases in brain-derived neurotrophic factor levels than nonresponders (44.77% [95% CI, 10.76% to 78.79%] vs −26.2% [95% CI, −58.53% to 6.12%]; *P* = .003). Furthermore, early decreases in serum interleukin 1 receptor antagonist in patients with metastatic ESCC and CRC were associated with longer progression-free survival (ESCC: not reached vs 2.1 months; hazard ratio, 0.19; 95% CI, 0.04 to 0.95; *P* = .04; CRC: not reached vs 2.1 months; hazard ratio, 0.06; 95% CI, 0.01 to 0.38; *P* < .001). Early increases in brain-derived neurotrophic factor levels in patients with metastatic GC were associated with longer progression-free survival (not reached vs 4.2 months; hazard ratio, 0.15; 95% CI, 0.03 to 0.84; *P* = .03).

**Conclusions and Relevance:**

In this study, baseline serum levels of monocyte chemoattractant protein 1, leukemia inhibitory factor, and cluster of differentiation 152 were associated with hyperprogressive metastatic gastrointestinal cancer among patients receiving ICB. An early decrease in serum interleukin 1 receptor antagonist levels in patients with metastatic ESCC or CRC and an early increase in serum brain-derived neurotrophic factor levels in patients with metastatic GC were better able to identify who would respond to ICB compared with microsatellite stability status or programmed cell death ligand 1 expression.

## Key Points

**Question:**

Are serological biomarkers associated with response to immune checkpoint blockade in patients with hyperprogressive metastatic gastrointestinal tract cancer?

**Findings:**

In this cohort study including 56 patients, baseline serum levels of monocyte chemotactic protein 1, leukocyte inhibition factor, and cluster of differentiation 152 were associated with hyperprogressive metastatic gastrointestinal cancer. Early changes in serum interleukin 1 receptor antagonist levels were associated with response to immune checkpoint blockade in patients with metastatic esophageal squamous cell carcinoma and colorectal cancer, and early changes in brain-derived neurotrophic factor levels were associated with response in patients with gastric cancer.

**Meaning:**

A panel of serological biomarkers may help oncologists identify patients with hyperprogressive disease before immune checkpoint blockade therapy and predict immune checkpoint blockade responses at an early stage, thereby providing an opportunity for early intervention.

## Teaser

This cohort study evaluates the association of serological biomarkers with the response to immune checkpoint blockade therapy in patients with metastatic gastrointestinal tract cancer.

## Introduction

Targeting the programmed cell death receptor 1/programmed cell death ligand 1 (PD-1/PD-L1) pathway has resulted in unprecedented advances in cancer immunotherapy.^1,2,3^ However, the overall response rate is only 10% to 30% in gastrointestinal (GI) tract cancer,^4^ and delayed clinical responses have also been observed with this treatment.^5^ Moreover, accumulating evidence indicates that GI tract cancers with different histopathological and biological features exhibit distinct behaviors, leading to different treatment responses.^6,7^ Therefore, it is necessary to establish biomarkers that can guide patient selection for immune checkpoint blockade (ICB) treatment.

However, few biomarkers are available to predict the response to ICB. Microsatellite instability–high (MSI-H) or DNA mismatch repair deficiency (dMMR) have been the only biomarkers approved by the US Food and Drug Administration for use of pembrolizumab to treat patients with unresectable or metastatic solid tumors.^8^Unfortunately, MSI-H and dMMR occur in only 5% or less^9,10,11^ of patients with metastatic GI tract cancers, who have a therapeutic response of approximately 40%.^12^ In a 2019 American Society of Clinical Oncology GI tract meeting,^13^ a PD-L1 combined positive score of 10 or more was considered a promising biomarker in advanced esophageal cancer treated with pembrolizumab. However, this population represented only approximately 30% of patients with esophageal cancer in the study, and the predictive value of PD-L1 expression still needs further investigation.^13^ Thus, most patients with metastatic GI tract cancer lack predictive markers for ICB treatment. Although various studies have shown that tumor mutational burden,^14^ microbiota,^15^ and tumor-infiltrating lymphocytes^16^ correlate with the efficacy of diverse anti-PD-1/PD-L1 drugs in non–small cell lung cancer and melanoma, the correlation between those biomarkers and the efficacy of PD-1/PD-L1 blockade in GI tract cancer is uncertain. In addition, between 9% and 29% of patients with solid tumors undergo rapid progression while receiving ICB therapy, which is described as hyperprogressive disease (HPD),^17,18^ and the prognosis for such patients is dismal. Moreover, to our knowledge, there is no predictive biomarker for HPD. Therefore, it is imperative to identify novel biomarkers that are associated with HPD and response to ICB in patients with metastatic GI tract cancer.

Correlations of systematic immunological factors, including serum growth factors, chemokines, cytokines, and soluble immune checkpoint molecules, with tumor burden and the microenvironment have been demonstrated, and they may also contribute to the efficacy of immunotherapy.^19,20,21^ However, the potential role of these serum protein levels in GI tract cancers has yet to be established. We examined the levels of 59 serum proteins using multiplexed bead immunoassays and investigated their associations with the clinical response to ICB treatment in patients with metastatic GI tract cancer.

## Methods

### Patients and Study Design

We reviewed all medical records of patients in the Department of Gastrointestinal Oncology, Peking University Cancer Hospital and Institute, Beijing, China, from August 1, 2015, to July 31, 2017, with a final follow-up date of January 1, 2018. Patients were eligible for inclusion if they received at least 1 cycle of any ICB treatment regardless of the agent’s target (ie, PD-1/PD-L1 or cytotoxic T-lymphocyte–associated protein 4). Treatment and sample collection details are provided in the eAppendix in the Supplement. This study was approved by the medical ethics committee of Peking University Cancer Hospital and was performed according to the principles of the Declaration of Helsinki.^22^ Written informed consent was obtained from all patients before inclusion in the study. This report follows the Strengthening the Reporting of Observational Studies in Epidemiology (STROBE) reporting guideline.

### Sample Collection and Multiplexed Bead Immunoassays

Serum samples were collected at baseline and during the first visit to the clinic 2 to 3 weeks after patients started treatment. Peripheral blood samples were obtained by venipuncture (10 mL, BD vacutainer blood collection tube; BD Biosciences) and centrifuged (1000*g*, 15 minutes) to isolate the serum. A total of 59 factors were simultaneously measured in serum samples using the ProcartaPlex Human Cytokine/Chemokine/Growth Factor Panel (Affymetrix, Inc) and the ProcartaPlexHuman Immuno-Oncology Checkpoint Panel (Affymetrix, Inc). A list of all serological factors and details are provided in the eAppendix in the Supplement.

### Statistical Analysis

Quantitative variables are presented as means and SDs or medians and interquartile ranges, and categorical variables are presented as proportions. The *t *test was used to analyze difference between 2 groups of normally distributed variables, and the Mann-Whitney *U* test was used for nonnormally distributed variables. The χ^2^ test was used to compare categorical variables. The correlations between serum protein levels and different variables were analyzed by Pearson correlation test or Spearman rank correlation test. Tree-based estimators were used to compute serum protein level importance, and irrelevant features with low weight were discarded. The computation was carried out in a Python environment. A receiver operating characteristic (ROC) curve was generated to evaluate the diagnostic accuracy of a protein. The area under the ROC curve (AUC) was used as a measure of discriminatory ability for the signature scores. The Youden index, a summary measure of the ROC curve,^23^ was used as an agnostic method for choosing an optimal cutoff value on the signature score to illustrate potential clinical usefulness. Progression-free survival and overall survival analyses were conducted with the Kaplan-Meier method and log-rank test. Statistical analyses were performed with SPSS version 21.0 (IBM Corporation). GraphPad Prism version 7.0 for Windows (GraphPad Software, Inc) was applied to analyze ROC curves. Data analysis was conducted from January 16, 2018, to September 1, 2018. *P* < .05 was considered statistically significant, and tests were 2-tailed.

## Results

### Patient Population

A total of 56 patients with GI tract cancers were included in the analysis (eFigure 1 in the Supplement). The baseline and treatment characteristics of the patients are shown in the Table. All patients had a confirmed objective response according to the Response Evaluation Criteria in Solid Tumors guideline version 1.1, with a response rate of 26.8% and a disease control rate of 46.4%.

**Table.** Characteristics of Patients

Characteristic	Patients, No. (%)	
	HPD	Non-HPD
Sex		
Male	3 (5.4)	36 (55.3)
Female	2 (3.5)	15 (26.8)
Age, y		
Median (IQR) [range]	51 (41-62) [31-63]	55 (49-63) [22-77]
<60	3 (5.4)	35 (66.7)
≥60	2 (3.5)	16 (33.3)
Original site		
Stomach	1 (1.8)	14 (25.0)
Esophagus	2 (3.5)	13 (23.2)
Colorectum	2 (3.5)	13 (23.2)
Other^a^	0	11 (19.6)
Histopathology		
Adenocarcinoma	2 (3.5)	29 (51.8)
Squamous carcinoma	1 (1.8)	13 (23.2)
Neuroendocrine tumor	2 (3.5)	6 (10.7)
Hepatocellular carcinoma	0	3 (5.4)
Treatment option		
Anti-PD-1 therapy	0	34 (60.7)
Anti-PD-L1 therapy	4 (7.1)	14 (25.0)
Anti-PD-L1 and anti-CTLA4 therapy	1 (1.8)	3 (5.4)
Lines of treatment		
2	3 (5.3)	26 (46.4)
≥3	2 (3.5)	25 (44.6)
Best response		
Complete response	0	1 (1.8)
Partial response	0	14 (25.0)
Stable disease	0	11 (19.6)
Progressive disease	5 (8.9)	25 (44.6)
Tumor MMR/MSI status		
dMMR/MSI-H	1 (1.8)	18 (32.1)
pMMR/MSS	3 (5.4)	23 (41.1)
NA	1 (1.8)	10 (17.9)
Tumor PD-L1 expression		
Positive	1 (1.8)	19 (33.9)
Negative	2 (3.5)	12 (21.4)
NA	2 (3.5)	20 (35.7)

Abbreviations: CTLA4, cytotoxic T-lymphocyte–associated protein 4; dMMR, DNA mismatch repair deficiency; HPD, hyperprogressive disease; IQR, interquartile range; MMR, mismatch repair; MSI, microsatellite instability; MSI-H, microsatellite instability–high; MSS, microsatellite stable; NA, not available; PD-1, programmed cell death receptor 1; PD-L1, programmed cell death ligand 1; pMMR, DNA mismatch repair proficiency.

^a^ Includes 4 patients with hepatic carcinoma, 6 with neuroendocrine tumors, and 1 with cholangiocarcinoma.

We observed HPD in 5 patients: 2 had adenocarcinomas, 2 had neuroendocrine carcinoma, and 1 had squamous cell carcinoma. Most patients with HPD (4 [80.0%]) were treated with PD-L1 blockade, and 1 patient with HPD (20.0%) was treated with PD-L1 blockade combined with cytotoxic T-lymphocyte–associated protein 4 blockade. Pseudoprogression was observed in 1 patient with colon adenocarcinoma. The first evaluation after immunotherapy showed progressive disease with a more than 25% initial increase in tumor burden and subsequent imaging evaluations that fulfilled the criteria for partial response.

### Identification of HPD Using Baseline Serum Levels

**Figure 1.**
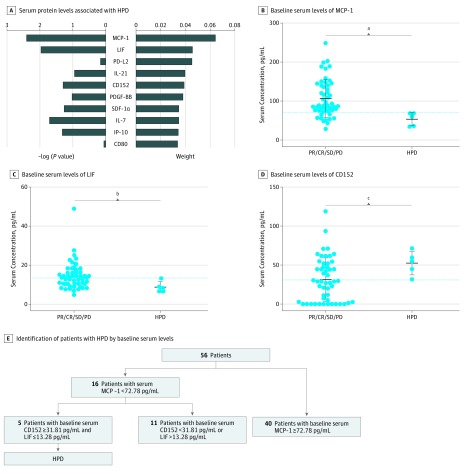
Contribution of Baseline Serum Protein Levels to Distinguish Hyperprogressive Disease (HPD) A, The weight factors for the baseline serum protein levels were ranked according to their ability to distinguish patients with HPD (n = 5) from those without HPD (n = 51). The −log (*P* value) and weight of the top 10 factors are shown. B-D, Baseline serum levels of monocyte chemotactic protein 1 (MCP-1), leukocyte inhibition factor (LIF), and cluster of differentiation 152 (CD152) in patients with HPD and patients without HPD. Dotted lines indicate cutoff values for the corresponding proteins. Center line represents the median value; upper and lower whiskers represent the 95th percentile and 5th percentile, respectively. E, All patients with HPD (5 of 56 [8.9%]) were identified by lower serum MCP-1 levels (<72.78 pg/mL) and LIF levels (≤13.28 pg/mL) and higher serum CD152 levels (≥31.81 pg/mL) at baseline. CD-80 indicates cluster of differentiation 80; CR, complete response; IL-21, interleukin 21; IL-7, interleukin 7; IP-10, interferon γ-induced protein 10; PD, progressive disease; PDGF-BB, platelet-derived growth factor, B subunits; PD-L2 programmed cell death ligand 2; PR, partial response; SDF-1α, stromal cell-derived factor 1α; and SD, stable disease. ^a^Cutoff for MCP-1 was 72.78 pg/mL, and *P *for comparison was .02. ^b^Cutoff for LIF was 13.28 pg/mL, and *P *for comparison was *.*01. ^c^Cutoff for CD152 was 31.81 pg/mL, and *P *for comparison was .03.

The contribution of each serum protein to distinguish patients with HPD (n = 5) and patients without HPD (n = 51) was assessed by multivariate analysis, and the their −log (*P* value) and weight of the top 10 factors were plotted (Figure 1A). The 5 most important factors distinguishing patients with HPD from those without HPD were concentrations of monocyte chemotactic protein 1 (MCP-1), leukocyte inhibition factor (LIF), programmed cell death ligand 2, interleukin 21, and cluster of differentiation 152 (CD152). In the 5 patients with HPD, median (SD) serum MCP-1 levels were significantly lower than those of the other 51 patients (53.4 [17.3] pg/mL vs 106 [48.4] pg/mL; *P* = .02) (Figure 1B), with an AUC of 0.87 (95% CI, 0.76-0.98; *P* = .007) (eFigure 2A in the Supplement). Next, we analyzed serum LIF, programmed cell death ligand 2, interleukin 21, and CD152 levels in 16 patients with lower MCP-1 levels at baseline and found that all patients with HPD had lower LIF levels (≤13.28 pg/mL) (Figure 1C) (eFigure 2B in the Supplement) and higher serum CD152 levels (≥31.81 pg/mL) (Figure 1D) (eFigure 2C in the Supplement) compared with patients without HPD. Thus, we identified all HPD patients (5 of 56 [8.9%]) using this panel of serum biomarkers (Figure 1E).

In addition, we found accelerated tumor growth in all 5 HPD patients, and HPD was associated with a worse outcome (eFigure 3 in the Supplement). The patients with HPD had a much shorter median progression-free survival (1.4 months vs 4.2 months; *P* < .001) and overall survival (3.6 months vs 11.4 months; *P* < .01) compared with patients without HPD.

### Correlation Between Baseline Serum Biomarkers and Response to ICB

**Figure 2.**
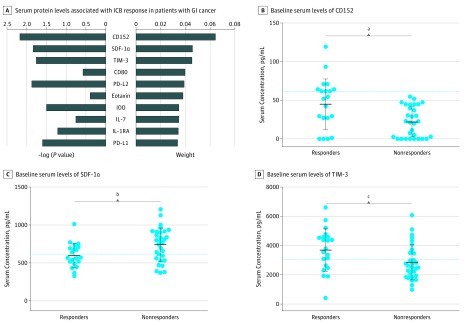
Contribution of Baseline Serum Protein Levels to Clinical Response in Patients With Gastrointestinal (GI) Tract Cancer Receiving Immune Checkpoint Blockade A, The weight factors for the baseline serum protein levels were ranked according to their ability to distinguish responders (n = 21) from nonresponders (n = 30) in patients with GI tract cancer but no hyperprogressive disease. The −log (*P* value) and weight of the top 10 factors are shown. B-D, Baseline serum levels of cluster of differentiation 152 (CD152), stromal cell-derived factor 1α (SDF-1α), and T-cell immunoglobulin and mucin domain 3 (TIM-3) in responders compared with nonresponders. Dotted lines indicate cutoff values for the corresponding proteins. The center line represents the median value, and the upper and lower whiskers represent the 95th percentile and 5th percentile, respectively. CD-80 indicates cluster of differentiation 80; IDO, indoleamine 2,3-dioxygenase; IL-1RA, interleukin 1 receptor antagonist; IL-7, interleukin 7; PD-L1, programmed cell death ligand 1; and PD-L2 programmed cell death ligand 2. ^a^Cutoff for CD152 was 61.78 pg/mL, and *P *for comparison was .003. ^b^Cutoff for SDF-1α was 772.20 pg/mL, and *P *for comparison was .02. ^c^Cutoff for TIM-3 was 3072.64 pg/mL, and *P *for comparison was .03.

To identify serum biomarkers associated with response to ICB therapy at baseline, we performed a multivariate analysis of potential factors for patients without HPD (n = 51). We identified CD152, stromal cell-derived factor 1α (SDF-1α), and T-cell immunoglobulin and mucin domain 3 (TIM-3) as the top 3 proteins, ranked by weight, that distinguished responders from nonresponders (Figure 2A).

Among responders, mean (SD) serum CD152 levels were significantly higher (44.9 [32.5] pg/mL vs 22.1 [19.7] pg/mL; *P* = .003), mean (SD) serum SDF-1α levels lower (600.8 [156.4] pg/mL vs 741.4 [219.2] pg/mL; *P* = .02), and mean [SD] serum TIM-3 levels higher (3706 [1464] pg/mL vs 2860 [1214] pg/mL; *P* = .03) than among nonresponders (Figure 2B-D). The AUC value for CD152 was 0.72 (95% CI, 0.57-0.87; *P* < .001); for SDF-1α, 0.70 (95% CI, 0.56-0.85; *P* = .02); and for TIM-3, 0.70 (95% CI, 0.55-0.85; *P* = .02) (eFigure 4 in the Supplement).

### Association of Early Changes in Serum Levels With Response to ICB

**Figure 3.**
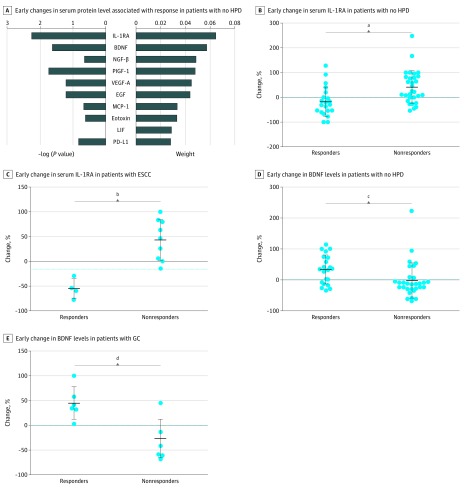
Association of Early Changes in Serum Interleukin 1 Receptor Antagonist (IL-1RA) and Brain-Derived Neurotrophic Factor (BDNF) Levels With Response to Immune Checkpoint Blockade A, The weight factors for early changes in serum protein levels from baseline to beginning of the second treatment cycle were ranked according to their ability to distinguish responders (n = 21) from nonresponders (n = 30) in patients without hyperprogressive disease (HPD). The −log (*P* value) and weight of the top 10 factors are shown. B-E, Dotted lines indicate cutoff values. The center line represents median value, and upper and lower whiskers represent 95th percentile and 5th percentile, respectively. The dots indicated serum concentration of patients. B-C, The early change of serum IL-1RA levels in responders and nonresponders in all 51 patients and patients with esophageal squamous cell carcinoma (ESCC), respectively. D-E, The early change of serum BDNF levels in responders and nonresponders in all 51 patients and patients with gastric cancer (GC). EGF indicates epidermal growth factor; HGF, hepatocyte growth factor; LIF, leukocyte inhibition factor; MCP-1, monocyte chemotactic protein 1; NGF-β, nerve growth factor β; PD-L1, programmed cell death ligand 1; PIGF-1, placenta growth factor 1; and VEGF-A, vascular endothelial growth factor A. ^a^Cutoff for change in IL-1RA among all patients was 0%, and *P *for comparison was .002. ^b^Cutoff for change in IL-1RA among patients with ESCC was −14.38%, and *P *for comparison was less than .001. ^c^Cutoff for change in BDNF among all patients was 0%, and *P *for comparison was .02. ^d^Cutoff for change in BDNF among patients with GC was 0%, and *P *for comparison was .003.

Next, we compared the differences in early serum protein level changes for patients without HPD, and the weight of the top 10 factors and their −log (*P* value) were plotted (Figure 3A). Early changes in serum interleukin 1 receptor antagonist (IL-1RA) and brain-derived neurotrophic factor (BDNF) levels were identified as the 2 most important factors distinguishing responders from nonresponders.

In 51 patients without HPD (91.1%), IL-1RA levels decreased more in responders than nonresponders (−18.79% [95% CI, −44.78% to 9.20%] vs 41.47% [95% CI, 16.64% to 66.29%]; *P* = .002) (Figure 3B). Similarly, in 13 patients with ESCC (23.2%), serum IL-1RA levels decreased more in responders than nonresponders (−55.02% [95% CI, −86.52% to −23.51%] vs 43.44% [95% CI, 11.93% to 74.96%]; *P* < .001) (Figure 3C). A similar result was also seen in patients with CRC (−35.82% [95% CI, −67.38% to −4.26%] vs 59.14% [95% CI, −72.34% to 190.6%]; *P* = .04) (eFigure 5A in the Supplement). Interestingly, serum IL-1RA levels also decreased from 52.13 pg/mL at baseline to 24.67 pg/mL on day 21 in a patient with metastatic CRC who presented with pseudoprogression.

However, early change in IL-1RA levels did not distinguish responders from nonresponders in 14 patients with GC (27.5%) (19.73% [95% CI −36.3% to 75.76%] vs 32.28% [95% CI −12.87% to 77.42%]; *P* = .67) (eFigure 5B in the Supplement). The AUC value for change in IL-1RA levels in all patients without HPD was 0.77 (95% CI, 0.64-0.91; *P* = .001); for patients with ESCC, 1.00 (95% CI, 1.00-1.00; *P* = .006); for patients with CRC, 0.93 (95% CI, 0.77-1.08; *P* = .013); and for patients with GC, 0.56 (95% CI, 0.24–0.88; *P* = .69) (eFigure 5C-F in the Supplement).

Second, we analyzed early changes in BDNF levels in each patient category. In all patients without HPD, BDNF levels tended to increase among responders and decrease among nonresponders (33.08% [95% CI, 11.9% to 54.26%] vs −2.22% [95% CI, −23.35% to 18.9%]; *P* = .02) (Figure 3D). In patients with GC, a significant difference was detected between responders and nonresponders (44.77% [95% CI, 10.76% to 78.79%] vs −26.21% [95% CI, −58.53% to 6.12%]; *P* = .003) (Figure 3E). However, early change in BDNF levels did not distinguish responders from nonresponders among patients with ESCC or CRC (ESCC: −5.07% [95% CI, −51.62% to 41.49%] vs −10.66% [95% CI, −31.88% to 10.56%]; *P* = .75; CRC: 40.53% [95% CI, −5.55% to 86.61%] vs −4.65% [95% CI, −49.47% to 40.17%]; *P* = .14) (eFigure 5G and eFigure 5H in the Supplement). The AUC value for change in BDNF level in patients without HPD was 0.72 (95% CI, 0.58-0.87; *P* = .007); for patients with ESCC, 0.56 (95% CI, 0.17-0.94; *P* = .76); for patients with CRC, 0.70 (95% CI, 0.40-1.00; *P* = .24); for patients with GC, 0.92 (95% CI, 0.75-1.08; *P* = .01) (eFigure 5I-L in the Supplement).

### Assessment of Predictive and Prognostic Values of Early Changes in IL-1RA and BDNF Levels in Patients With GI Tract Cancer

**Figure 4.**
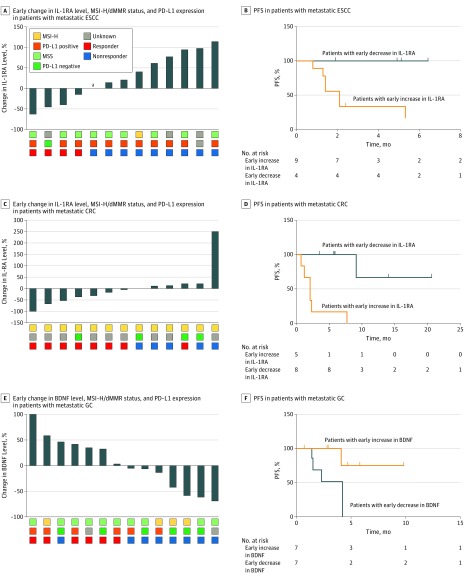
Early Changes in Interleukin 1 Receptor Antagonist (IL-1RA) and Brain-Derived Neurotrophic Factor (BDNF) Levels and Progression-Free Survival (PFS) in Patients With Gastrointestinal Tract Cancer CRC indicates colorectal cancer; dMMR, DNA mismatch repair deficiency; ESCC, esophageal squamous cell carcinoma; GC, gastric cancer; MSI-H, microsatellite instability–high; MSS, microsatellite stable; and PD-L1, programmed cell death ligand 1. ^a^Of 56 patients, 1 patient had no change to IL-1RA levels.

We further investigated MSI-H/dMMR status in patients with ESCC or CRC and PD-L1 expression in patients with GC, comparing them with early changes in IL-1RA and BDNF levels (Figure 4A, Figure 4C, and Figure 4E). Of 13 patients with ESCC, 4 (30.8%) were responders and 9 nonresponders (69.2%). Of the responders, 0 of 3 (data for 1 not available) had MSI-H/dMMR tumors, 3 of 4 (75.0%) had PD-L1–positive tumors, and all had serum IL-1RA levels that decreased by at least 14.38%. Of the nonresponders, 1 of 7 (14.3%; data for 2 not available) had dMMR/MSI-H tumors, 8 of 8 (data for 1 not available) had PD-L1–positive tumors, and all had a decrease less than 14.38% or an increase in serum IL-1RA levels (Figure 4A).

Of 13 patients with CRC, 8 were responders (61.5%) and 5 nonresponders (38.5%). Of the responders, all had MSI-H/dMMR tumors, 0 of 2 (data for 6 not available) had PD-L1–positive tumors, and 7 of 8 (87.5%) showed decreased serum IL-1RA levels. Of the nonresponders, 5 of 5 had MSI-H/dMMR tumors, 0 of 2 (data for 3 not available) had PD-L1–positive tumors, and all showed either no change or an increase in serum IL-1RA levels (Figure 4C).

Of 14 patients with GC, 6 were responders (42.9%) and 8 nonresponders (57.1%). Of the responders, 1 of 6 (16.7%) had MSI-H/dMMR tumors, 4 of 5 (80.0%; data for 1 not available) had PD-L1–positive tumors, and all showed increased serum BDNF levels. Of the nonresponders, 3 of 8 (37.5%) had MSI-H/dMMR tumors, 2 of 7 (28.6%; data for 1 not available) had PD-L1–positive tumors, and 7 of 8 (87.5%) showed decreased serum BDNF levels (Figure 4E).

Progression-free survival was significantly longer in patients with metastatic ESCC presenting with an early decrease in serum IL-1RA level compared with patients showing an early increase (not reached vs 2.1 months; hazard ratio, 0.19; 95% CI, 0.04-0.95; *P* = .04) using the same stratification cutoff value as in the response assessment (decrease ≥14.38%) (Figure 4B). Similar results were seen among patients with CRC (not reached vs 2.1 months; hazard ratio, 0.06; 95% CI, 0.01-0.38; *P* < .001) (Figure 4D). Progression-free survival was significantly longer in patients with metastatic GC presenting with an early increase in serum BDNF levels compared with patients showing an early decrease (not reached vs 4.2 months; hazard ratio, 0.15; 95% CI, 0.03-0.84; *P* = .03) (Figure 4F).

## Discussion

This study evaluated the baseline levels and early changes in serum proteins to explore biomarkers associated with response to ICB in patients with metastatic GI tract cancer. Using baseline serum MCP-1, LIF, and CD152 levels, we were able to identify all patients with HPD (5 of 56 [8.9%]) with metastatic GI tract cancer. Early changes in serum IL-1RA were associated with response to ICB in patients with metastatic ESCC or CRC, and early changes in BDNF levels were associated with response to ICB in patients with GC (eFigure 6 in the Supplement).

Hyperprogressive disease is a unique anti-PD-1/PD-L1–induced response pattern, characterized by accelerated tumor growth and shorter progression-free survival and overall survival.^17^ Therefore, it is a priority to identify patients with HPD before starting ICB treatment. However, this study found no predictive biomarker for HPD, and the underlying mechanism of HPD remains unclear. Other studies have shown that *MDM2*/*MDM4 *amplification can inhibit the p53 tumor suppressor^24^ and is correlated with HPD following ICB treatment.^25^ In this study, patients with HPD showed lower baseline MCP-1 levels, lower baseline LIF levels, and higher baseline CD152 levels. We speculate that these proteins are regulated by p53.^26,27,28^ Thus, this panel of serum biomarkers may be alternative indicators of *MDM2*/*MDM4* amplification in tumor cells, which may be a predictive biomarker for HPD.

Next, we tried to identify predictive biomarkers for responders receiving ICB and found that baseline serum CD152, SDF-1α, and TIM-3 levels were most associated with response to ICB treatment. Previous studies have shown that higher CD152 and TIM-3 levels indicate a lymphocyte-inflamed tumor microenvironment,^29,30^ and SDF-1α may induce migration of monocytes, which may be correlated with the response to ICB.^31,32^ However, none of the ROC curves in this study had good sensitivity or specificity.

Owing to pseudoprogression’s delayed kinetic and atypical response patterns,^33^ a traditional imaging evaluation cannot be used to distinguish progressive disease from pseudoprogression, which presents with increased tumor size on computed tomography or magnetic resonance imaging scans before regression.^5^ Therefore, researchers have switched their focus to dynamic parameters to predict the response to ICB. Sanmamed et al^34^ reported that changes in serum interleukin 8 levels are associated with the response to ICB in patients with melanoma and non–small cell lung cancer. However, interleukin 8 is a member of the CXC chemokine family and was originally identified as a chemotactic factor of neutrophils that play an important role in the tumor microenvironment, including in non–small cell lung cancer.^35^ Monocyte/macrophage infiltration is more common than neutrophils in esophageal cancer and CRC,^36^ which is consistent with the low concentrations of serum interleukin 8 observed in this study. Interleukin 1 receptor antagonist is a member of the IL-1 family and is secreted mainly by cells of the monocyte/macrophage lineage.^37^ Moreover, the serum IL-1RA level is correlated with local characteristics of multiple tumors, including CRC,^38,39^ and could be a reflection of anti-IL-1 signaling–mediated protection against tumors. Our results suggest that a decrease in serum IL-1RA level was associated with a better treatment response, which may point to inhibition of IL-1RA signaling following ICB treatment. Furthermore, the early change in serum IL-1RA levels was also associated with the true response of a patient with pseudoprogression. The results in this study show that early changes in serum IL-1RA level might be a useful biomarker to monitor the response during ICB treatment in patients with metastatic ESCC or CRC.

Interestingly, we also found that an increase in serum BDNF level was associated with a better response in patients with GC. Initially identified as a protein secreted by neurons, BDNF affects nervous system development. Previous reports^40,41^ have demonstrated significant correlations of elevated BDNF or tropomyosin-related kinase B pathway-axis expression with disease progression and poor prognosis in patients with GC. In addition, other studies^42,43,44,45^ have suggested that BDNF is produced by several types of cancer. However, the correlation between changes in BDNF levels and the response to ICB treatment has not been investigated to our knowledge. Based on the results from this study, early changes in BDNF level could serve as a biomarker to monitor the response to ICB in patients with GC.

Compared with MSI-H/dMMR status and PD-L1 expression, changes in serum IL-1RA and BDNF levels were superior in identifying patients with metastatic ESCC or CRC and GC, respectively, who would respond to ICB therapy. Unfortunately, we could not draw a conclusion regarding patients with neuroendocrine carcinoma or other cancers because of the small number of patients.

These results suggest that early changes in IL-1RA and BDNF levels are associated with progression-free survival in patients with metastatic GI tract cancer. Owing to the short follow-up period, median overall survival was not reached in those patients. In other studies,^46,47^ numerous blood parameters were investigated as potential inflammatory biomarkers, including elevated serum IL-6 and CD152 concentrations, which are correlated with poor outcomes in patients with cancer. Based on our results, these serum protein levels seem to be not only predictive but also prognostic biomarkers in patients treated with ICB.

### Limitations

This study is subject to the limitations in observational retrospective studies. For example, a high rate of patients had unknown PD-L1 status and MSI-H/dMMR status, and we usually did not have enough samples to reanalyze those biomarkers. In addition, the ICBs in this study were from different pharmaceutical companies, which might cause some bias in the results.

## Conclusions

In summary, this study explored the levels of multiple serum proteins in patients with metastatic GI tract cancer receiving ICB. The results suggested that baseline serum levels of MCP-1, LIF, and CD152 were associated with HPD. Compared with MSI-H/dMMR status and PD-L1 expression, an early decrease in IL-1RA level was better at identifying responders to ICB among patients with metastatic ESCC or CRC. Similarly, an early increase in BDNF level was better at identifying responders among patients with metastatic GC compared with dMMR/MSI-H or PD-L1 status. These results support the hypothesis that baseline and early changes in these serum protein levels could contribute to predicting patients with HPD and selecting patients who may benefit from ICB. However, further prospective studies are needed to confirm these findings.

## Notes

Supplement. **eAppendix. **Additional Methods **eFigure 1. **Flow Diagram of Patient Selection **eFigure 2. **ROC Curve Analysis of Baseline MCP-1, LIF, and CD152 to Distinguish HPD **eFigure 3. **Worse Outcome Associated With HPD Among Patients With GI Tract Cancer Treated With ICB **eFigure 4. **ROC Curve Analysis of Baseline MCP-1, LIF, and CD152 in Responders Compared With Nonresponders **eFigure 5. **Early Changes in Serum Protein Levels Associated With the Response to ICB **eFigure 6. **A Schematic Diagram of Serum Protein Levels Reflecting and Predicting the Response of GI Tract Cancer to ICB
