# Supplementary material for: Serological Markers Associated With Response to Immune Checkpoint Blockade in Metastatic Gastrointestinal Tract Cancer
Source: JAMA Netw Open. 2019 Jul 24;2(7):e197621. doi: 10.1001/jamanetworkopen.2019.7621 (PMC6659353; doi:10.1001/jamanetworkopen.2019.7621)
Supplement: Supplement. — eAppendix. Additional Methods eFigure 1. Flow Diagram of Patient Selection eFigure 2. ROC Curve Analysis of Baseline MCP-1, LIF, and CD152 to Distinguish HPD eFigure 3. Worse Outcome Associated With HPD Among Patients With GI Tract Cancer Treated With ICB eFigure 4. ROC Curve Analysis of Baseline MCP-1, LIF, and CD152 in Responders Compared With Nonresponders eFigure 5. Early Changes in Serum Protein Levels Associated With the Response to ICB eFigure 6. A Schematic Diagram of Serum Protein Levels Reflecting and Predicting the Response of GI Tract Cancer to ICB [file jamanetwopen-2-e197621-s001.pdf]

Supplementary Online Content

Lu Z, Zou J, Hu Y, et al. Serological markers associated with response to immune checkpoint blockade in metastatic gastrointestinal tract cancer. *JAMA Netw Open*. 2019;2(7):e197621. doi:10.1001/jamanetworkopen.2019.7621

eAppendix. Additional Methods

eFigure 1. Flow Diagram of Patient Selection

eFigure 2. ROC Curve Analysis of Baseline MCP-1, LIF, and CD152 to Distinguish HPD

eFigure 3. Worse Outcome Associated With HPD Among Patients With GI Cancer Treated With ICB

eFigure 4. ROC Curve Analysis of Baseline MCP-1, LIF, and CD152 in Responders Compared With Nonresponders

eFigure 5. Early Changes in Serum Protein Levels Associated With the Response to ICB

eFigure 6. A Schematic Diagram of Serum Proteins Reflecting and Predicting the Response of GI Cancer to ICB

This supplementary material has been provided by the authors to give readers additional information about their work.

© 2019 Lu Z et al. *JAMA Network Open.*

eAppendix 1. Additional Methods

Supplementary Methods

Patients and study design

Medical records from consecutive patients with malignant tumors of the digestive system who were treated with a programmed cell death-1 (PD-1)/program death ligand 1 (PD-L1) inhibitor (alone or combined with a CTLA-4 inhibitor) at Peking University Cancer Hospital were collected retrospectively (NCT02825940, NCT02978482, NCT02915432, NCT03167853, and CTR20160872). The patient characteristics were extracted from medical records. Details of onset, treatment, and efficacy were analyzed. The pathological and imaging results of all cases were reviewed retrospectively by two pathologists and two radiologists, respectively. The follow-up date was January 1, 2018.

Tumor burden was measured in all patients by imaging studies or physical examinations according to the modified RECIST v1.1. Patients were stratified and analyzed according to the presence of a response, progression, pseudoprogression, and HPD, as assessed by the investigators. Best response was defined as the best objective response [complete response (CR), partial response (PR), stable disease (SD), or progressive disease (PD)] assessed from the first day of treatment to progression, death, or the last follow-up. Responders were defined as patients who had the best overall response of CR, PR, or SD that persisted for ≥ 24 weeks. Pseudoprogression was defined as a ≥25% increase in the size of target tumor lesions or the appearance of new lesions that did not represent true tumor progression and were not confirmed as PD by subsequent imaging assessments. The tumor growth kinetics (TGK) of immunotherapy and TGK pre-immunotherapy were collected, and the TGK ratio was calculated. HPD was defined as a TGK ratio ≥ 2 (19). Progression-free survival was defined as the time from the start of treatment to documented evidence of true PD or death. Overall survival was defined as the time from treatment initiation to death from any cause.

Sample collection and storage

Serum samples were collected at baseline and during the first visit to the clinic (2–3 weeks) after starting treatment. Peripheral blood samples were obtained by venipuncture (10 mL; BD Vacutainer blood collection tube) and centrifuged (1000 x g, 15 min) to isolate the serum. The sera were sub-packed in multiple aliquots and stored in −80°C freezers, which were monitored, and activity was recorded in quality-controlled tissue banks that followed standard operating procedures. No freeze–thaw cycles were performed before analysis.

Quantification of serum proteins using multiplexed bead immunoassays

The panel of serum proteins used consists of two parts: part I, ProcartaPlex Human Cytokine/Chemokine/Growth Factor Panel (Affymetrix, Inc., Santa Clara, CA, USA), including IFN-γ, IL-12p70, IL-13, IL-1β, IL-2, IL-4, IL-5, IL-6, TNF-α, GM-CSF, IL-18, IL-10, IL-17A, IL-21, IL-22, IL-23, IL-27, IL-9, IFN-α, IL-31, IL-15, IL-1α, IL-1RA, IL-7, TNF-β, eotaxin, GRO-α, CD152, IP-10, MCP-1, MIP-1α, MIP-1β, SDF-1α, RANTES, NGF-β, BDNF, EGF, FGF-2, HGF, LIF, PDGF-BB, PlGF-1, SCF, VEGF-A, and VEGF-D; part II, ProcartaPlex Human Immuno-Oncology Checkpoint Panel (Affymetrix), including BTLA, GITR, HVEM, IDO, LAG-3, PD-1, PD-L1, PD-L2, TIM-3, LIF, CD80, CD137, CD27, and CD152.

Assays were performed in 96-well filter plates according to the manufacturer’s instructions. Briefly, reagents including 1× wash buffer and 1× universal assay buffer were prepared. Microsphere beads coated with monoclonal antibodies against the different target analytes were added to the wells. Standards and samples were pipetted into the wells and incubated with shaking at 500 rpm for 60–120 min at room temperature. The wells were washed using a handheld magnetic plate washer (eBioscience, Inc., San Diego, CA, USA), and a mixture of biotinylated detection antibodies was added. After a 30 min incubation at 500 rpm and room temperature, streptavidin conjugated to the fluorescent protein R-phycoerythrin was added to the beads and incubated for 30 min at 500 rpm and room temperature. After washing to remove the unbound reagents, reading buffer (Affymetrix) was added to the wells, and the beads were analyzed using the Luminex MAGPIX® instrument (Luminex Co., Austin, TX, USA).

The Luminex MAGPIX® monitors the spectral properties of the beads to distinguish the different analytes while simultaneously measuring the amount of fluorescence associated with R-phycoerythrin, which is reported as the

© 2019 Lu Z et al. *JAMA Network Open.*

median fluorescence intensity. The concentrations of the unknown samples (antigens in serum samples) were estimated from a standard curve using a 5PL algorithm and the ProcartaPlex Analyst 1.0 software (eBioscience) and are expressed in pg/mL after adjusting for the dilution factor. The concentration ranges of each biomarker were analyzed. The change in the biomarker concentration was calculated as follows:

Change in biomarker = $(C_{\text{after treatment}} - C_{\text{baseline}})/C_{\text{baseline}} \times 100\%$, where $C_{\text{after treatment}}$ is the concentration of the biomarker after an immunotherapy cycle, and C_{baseline} is the concentration of the biomarker at baseline.

Immunohistochemical (IHC) staining for PD-L1

Primary tumor specimens were obtained from patients with metastatic GI cancer before receiving checkpoint inhibitor blockade therapy at Beijing Cancer Hospital. Formalin-fixed paraffin-embedded tissue blocks were prepared and sectioned at 4 μm for IHC. The immunohistochemically stained tissue sections were scored separately by two pathologists blinded to the clinicopathological parameters. IHC staining for anti-PD-L1 (rabbit, clone SP142, 1:100; Spring Bioscience, CA, USA) was annotated within intratumoral areas. To evaluate PD-L1 expression, three fields of view (FOVs) in darkly stained areas were selected, and the percentages of cancer cells and immune cells stained by the anti-PD-L1 antibody in each FOV were measured under a microscope at 400×. Expression of PD-L1 was designated as positive, when ≥ 1% of the tumor/stromal cells were positive.

MMR/MSI Testing

Formalin-fixed paraffin-embedded (FFPE) sections were collected for IHC staining. To determine the microsatellite stability status, mutL homolog 1 (MLH1), mutS homolog 2 (MSH2), mutS homolog 6 (MSH6) and PMS1 homolog 2 (PMS2) were stained by using the following monoclonal antibodies: MLH1 (1:60; Clone ES05, Gene Tech, Inc., South San Francisco, CA, USA), MSH2 (1:40; Clone 25D12, Gene Tech), MSH6 (1:50; EP49, Gene Tech) and PMS2 (1:40; Clone EP51, Gene Tech). The complete loss of expression of one or more protein was considered as dMMR.

In some cases, MSI status was calculated using a single multiplex PCR, which assesses five microsatellite loci (BAT-25, BAT-26, D2S123, D5S346, and D17S250) recommended by the 1997 NCI-sponsored MSI workshop (1). For interpretation, instability at more than one locus was defined as MSI-H, instability at a single locus was defined as low MSI (MSI-L), and no instability at any locus was defined as MSS (2).

1. Boland CR, et al. A National Cancer Institute Workshop on Microsatellite Instability for cancer detection and familial predisposition: development of international criteria for the determination of microsatellite instability in colorectal cancer. Cancer Res. 1998;58(22):5248-57.

2. Jang M, et al. Microsatellite instability test using peptide nucleic acid probe-mediated melting point analysis: a comparison study. BMC Cancer. 2018;18(1):1218.

eFigure 1. Flow Diagram of Patient Selection

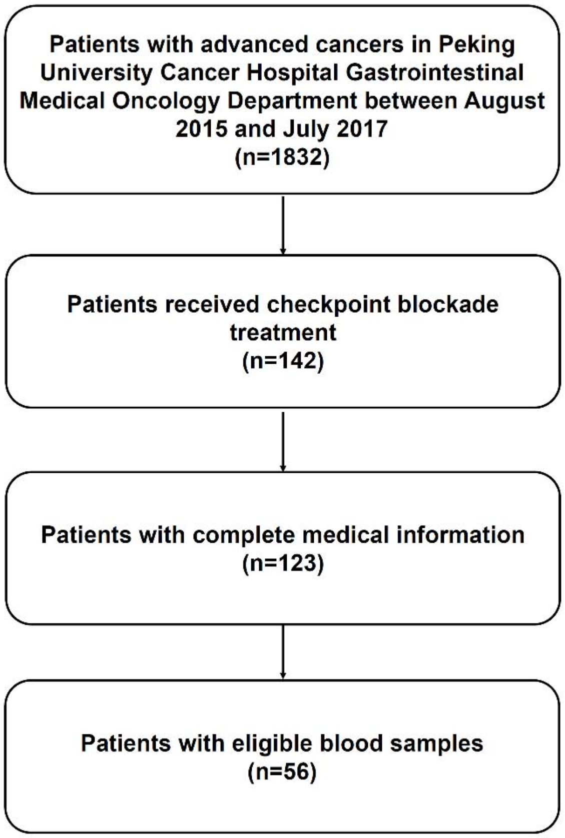

© 2019 Lu Z et al. *JAMA Network Open.*

eFigure 2. ROC Curve Analysis of Baseline MCP-1, LIF, and CD152 to Distinguish HPD

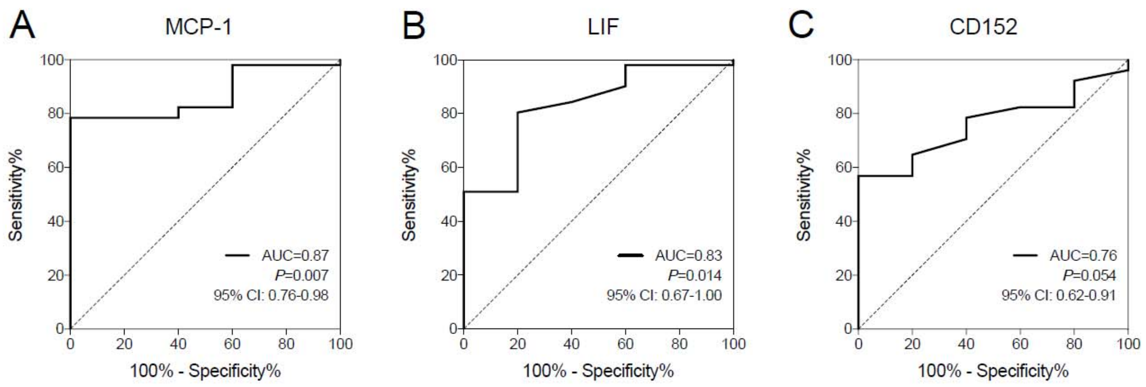

(A) ROC curve analysis of MCP-1 (A), LIF(B) and CD152(C) in HPD and non-HPD patients.

© 2019 Lu Z et al. *JAMA Network Open.*

eFigure 3. Worse Outcome Associated With HPD Among Patients With GI Cancer Treated With ICB

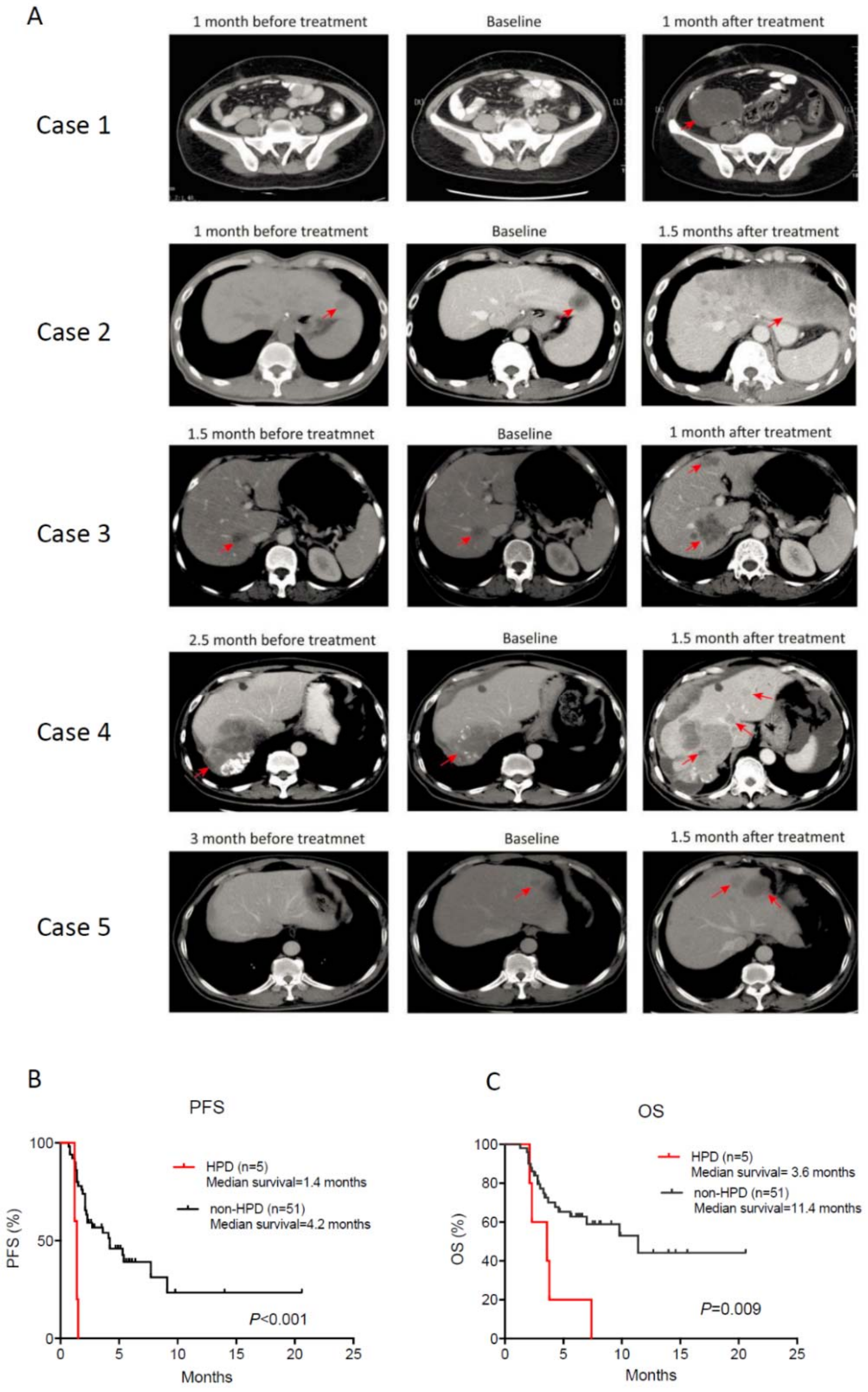

(A) Computed tomography (CT) images of the five HPD patients. CT scans were obtained at different time points; arrows indicate the tumor sites. Kaplan–Meier curves for PFS (B) and OS (C) were stratified by HPD and non-HPD.

© 2019 Lu Z et al. *JAMA Network Open.*

eFigure 4. ROC Curve Analysis of Baseline MCP-1, LIF, and CD152 in Responders Compared With Nonresponders

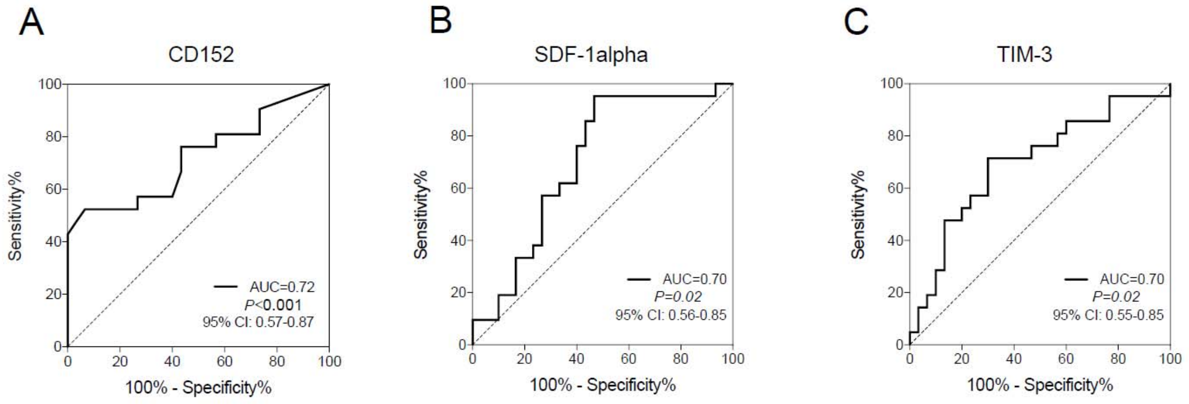

ROC curve analyses of early changes in serumCD152 (A), SDF-1 α (B) and TIM3 (B) in responders compared with non-responders .

© 2019 Lu Z et al. *JAMA Network Open.*

eFigure 5. Early Changes in Serum Protein Levels Associated With the Response to ICB

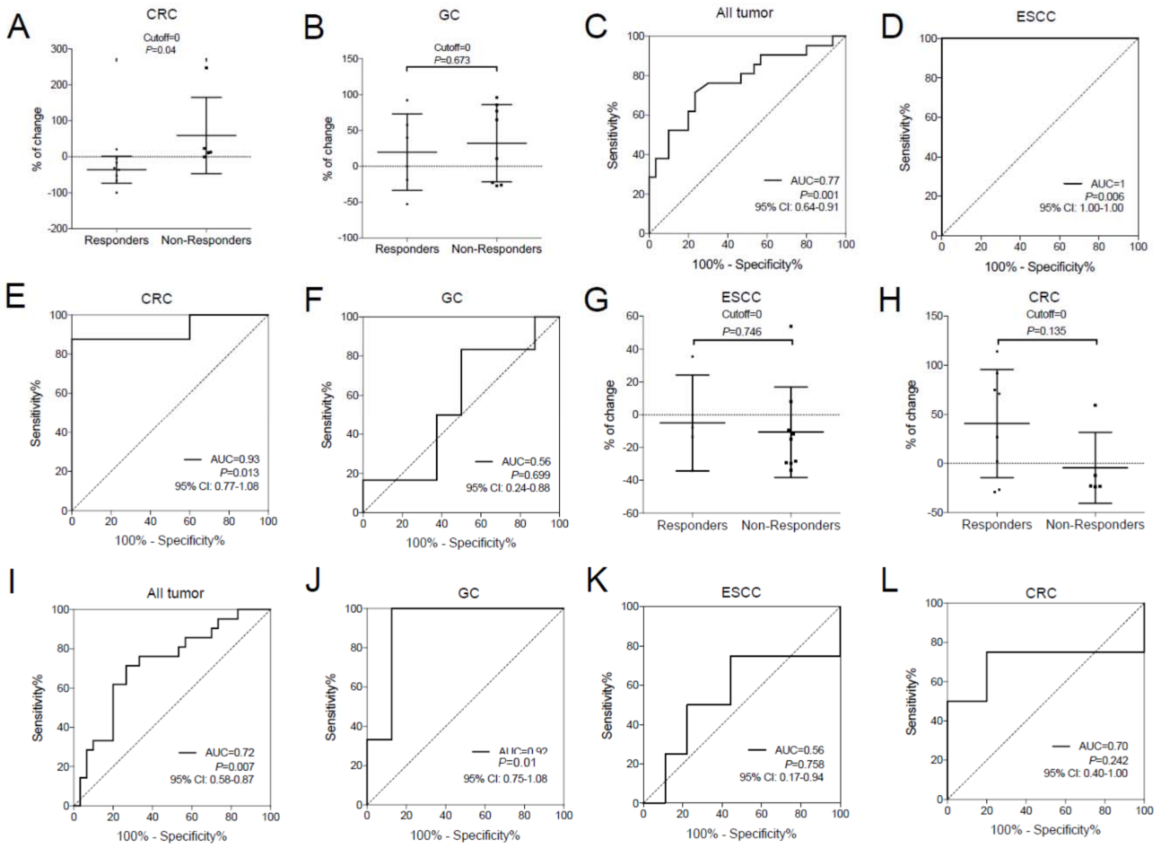

The early change of serum IL-1RA levels in responders and non-responders in CRC (A) and GC(B). The ROC curve of early change of serum IL1RA levels in responders and non-responders in all 51 patients (C), ESCC (D), CRC (E) and GC (F). The early change of serum BDNF levels in responders and non-responders in ESCC (G) and GC(H)Dotted lines indicate cutoff values of the corresponding proteins. The ROC curve of early change of serum BDNF evels in responders and non-responders in all 51 patients (I), GC (J), ESCC (K) and CRC (L). The center line, upper and lower whiskers represent the median value, 95th percentile and 5th percentile. The dots indicated serum concentration of patients.

© 2019 Lu Z et al. *JAMA Network Open.*

eFigure 6. A Schematic Diagram of Serum Proteins Reflecting and Predicting the Response of GI Cancer to ICB

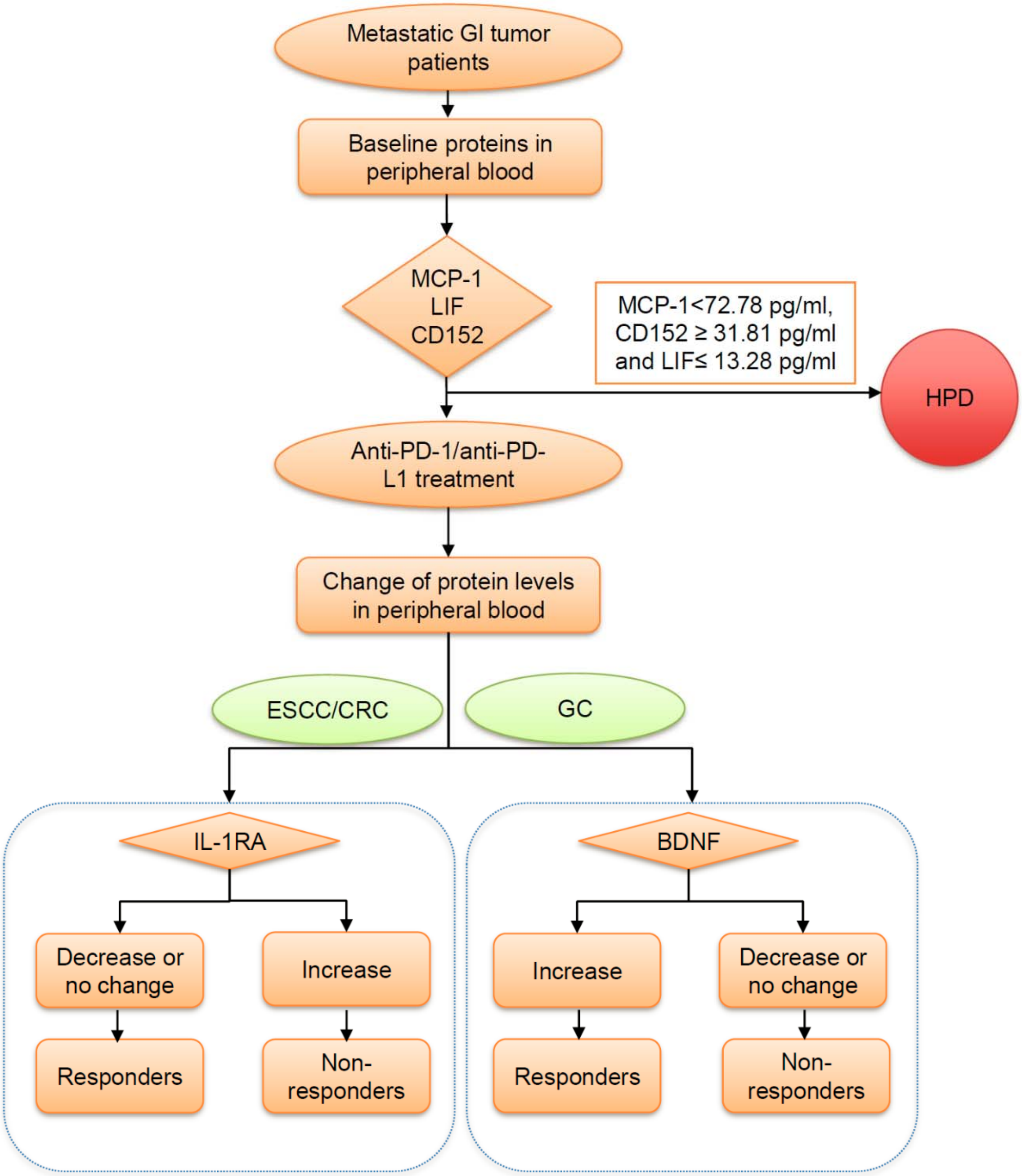

Baseline and early changes in serum protein levels reflected the responses of patients with metastatic GI cancer receiving checkpoint blockade therapy.

© 2019 Lu Z et al. *JAMA Network Open.*
